# Is There an Association Between Metformin Exposure and Frailty?

**DOI:** 10.1177/2333721420924956

**Published:** 2020-06-15

**Authors:** Dhanya Baskaran, Raquel Aparicio-Ugarriza, Juliana Ferri-Guerra, Raneem Milyani, Hermes Florez, Jorge G. Ruiz

**Affiliations:** 1 Miami VAHS Geriatric Research Education and Clinical Center (GRECC), FL, USA; 2 Miller School of Medicine, University of Miami, FL, USA; 3 Jackson Health System, Miami, FL, USA

**Keywords:** frailty, metformin, exposure, diabetic, veterans

## Abstract

Frailty is a state of vulnerability to stressors resulting in higher morbidity, mortality, and utilization in older adults. Frailty and type 2 diabetes mellitus share similar pathophysiological mechanisms which metformin may target. The purpose of this study was to determine whether exposure to metformin is associated with frailty in veterans. This is a cross-sectional study of veterans 65 years and older with type 2 diabetes who were screened for frailty between January 2016 and August 2017. We constructed a 44-item Frailty Index including multiple variables using a deficit accumulation framework. After adjustment for covariates, the association was calculated using binomial logistic regression models with frailty status as the outcome variable, and metformin exposure as the independent variable. Patients were 98.3% male and 56.7% White with a mean age of 72.9 (*SD* = 6.8) years. The proportion of robust, prefrail and frail patients was 2.9% (*n* = 22), 46.7 % (*n* = 356) and 50.5% (*n* = 385), respectively. In binomial logistic regression, exposure to metformin was associated with lower risk for frailty, adjusted odds ratio (OR) = .55 (95% confidence interval [CI] = .39–.77), *p* ≤ .001. This study shows that exposure to metformin was associated with lower risk for frailty in community-dwelling veterans.

## Introduction

Frailty is a state of vulnerability to stressors, characterized by multisystem impairments which may cause a reduction of intrinsic reserve and may result in disability, higher morbidity, mortality, and increased health care utilization in older adults (Fried et al., 2001; Rockwood & Mitnitski, 2007). Research evidence suggests that type 2 diabetes is an independent risk factor for frailty, but that frailty may also be an independent risk factor for type 2 diabetes, indicating a bidirectional relationship (Garcia-Esquinas et al., 2015; Sinclair et al., 2018). Frailty and diabetes share some of the same pathophysiological mechanisms: insulin resistance, chronic low-grade inflammation, oxidative stress, stem cell dysfunction, mitochondrial dysfunction, and sarcopenia (Abdelhafiz et al., 2016; Garcia-Esquinas et al., 2015; Morley et al., 2014; Sinclair & Rodriguez-Manas, 2016).

Comorbid medical and mental conditions often coexist in both frailty and type 2 diabetes, including but not limited to obesity, cardiovascular disease, sleep, depression, and cognitive impairment (Abdelhafiz & Sinclair, 2019; Morley et al., 2014). The shared pathophysiological mechanisms in both frailty and type 2 diabetes may benefit from interventions that jointly target these pathways.

Metformin has multiple systemic effects which are independent of its role in glucose control as an insulin sensitizer in patients with type 2 diabetes. Metformin may be beneficial in individuals with frailty through its effects in pathways common to both frailty and type 2 diabetes. Metformin displays multiple actions in multiple tissues and organs including anti-inflammatory and antioxidant effects, activation of the AMP-activated protein kinase (AMPK), effects on mitochondrial decline, inhibition of the mechanistic target of rapamycin (mTOR) pathway, effects on cellular senescence, autophagy, and reduction of glycation products (Barzilai et al., 2016; Piskovatska et al., 2019; Rena et al., 2017). Metformin’s pleiotropic effects on these mechanistic pathways, tissues, and organs may potentially contribute to the prevention or amelioration of the frailty state, which is characterized by multisystem dysfunction in older adults.

There is a dearth of research that examines the effect of metformin on preventing or treating frailty. As randomized trials evaluating the role of metformin on frailty may not be ethically feasible, additional observational studies are necessary to further examine the potential benefits of metformin for the prevention and treatment of frailty in patients with diabetes. Two observational studies looked at the effects of metformin in older adults with diabetes. In a cohort study, metformin compared with sulfonylurea-reduced mortality in older veterans with diabetes, but its benefits were also observed in nonfrail individuals (Wang et al., 2014). In another cohort of older male veterans with diabetes, metformin reduced the incidence of chronic conditions commonly associated with frailty, potentially explaining a pathway for lower mortality (Wang et al., 2017). An ongoing randomized controlled trial is investigating the use of metformin to prevent frailty in individuals with prediabetes (Espinoza et al., 2020), but there are no observational studies that have looked at whether metformin exposure is associated with frailty in older adults with diabetes. Both frailty (Orkaby et al., 2019) and diabetes (Liu et al., 2017) are more highly prevalent in the veterans rather than the general U.S. population. Diabetes (Benjamin et al., 2015) and frailty (Chang et al., 2018) are both independently associated with a higher risk for all-cause hospitalizations in older adults. Furthermore, mortality is also higher in frailty and diabetes. In veterans, the concurrence of frailty and diabetes may further increase the effects of individual conditions on clinical outcomes that may lead to higher health care utilization and mortality (Ferri-Guerra et al., 2020).

Although ongoing randomized controlled trials are investigating the use of metformin to prevent the development of frailty in individuals with prediabetes (Espinoza et al., 2020), trials in patients with established type 2 diabetes and frailty may not be feasible. The purpose of this study was to determine in a sample of older veterans with type 2 diabetes the cross-sectional association of metformin exposure with frailty. We hypothesized that metformin exposure will be independently and negatively associated with frailty after adjustment for factors known to be associated with this syndrome.

## Methods

### Design and Participants

The present research is a retrospective, cross-sectional study of community-dwelling veterans aged 65 years and older who were receiving outpatient care at a veterans affairs (VA) facility between January 2016 to August 2017 and have an established and confirmed diagnosis of diabetes mellitus. We then conducted a retrospective electronic health record review to determine the frailty status of participants and its association with metformin exposure. We obtained an exempted status as a quality improvement project from our institutional review board for this retrospective chart review.

#### Measures

We obtained sociodemographic information including age, gender, race, ethnic group, and median household income. We used the 5-Digit ZIP Code Tabulation Area (ZCTA) and median household income over the past 12 months (in 2011 inflation-adjusted dollars) by racial group from the U.S. Census Bureau, 2007 to 2011, to determine differences in median household income.

#### Metformin exposure

From data obtained from pharmacy records in the VA electronic health record and corporate data warehouse (CDW), exposure to metformin was determined. Only those individuals currently on metformin regardless of dosing and frequency of administration were included. Individuals who took metformin in the past were considered not exposed to metformin. We did not include the duration of metformin exposure. Patients were categorized as “No exposure” (code as 0) and “Yes exposure” (code as 1).

#### Frailty

A Frailty Index (FI) was generated from data obtained from the VA electronic health record and CDW. The FI was based on the deficit accumulation model of frailty and calculated as a proportion of the number of factors (sociodemographic, medical and psychological conditions, laboratory tests, number of medications, blood pressure, body mass index (BMI), and activities of daily living) present in over a total of 44 factors (see Supplementary Materials). A FI was calculated for each subject. At least 30 of 44 items were needed to calculate the FI and be included in the study. The patients were stratified as robust (FI is ≤ 0.10), prefrail (FI between 0.10 and 0.21) or frail (FI is ≥ 0.21) (Searle et al., 2008).

### Data Analysis

Baseline characteristics are presented as frequency (percent) for categorical variables and as mean ± standard deviation (*SD*) for normally distributed continuous variables. We reported descriptive statistics of age, marital status, race, ethnicity, and median household income in the previous year. We also conducted subgroup analyses, excluding patients with creatinine, number of medications, BMI, use of metformin, insulin and sulfonylureas, and age-adjusted Charlson Comorbidity Index (CCI). All the continuous variables showed no-normal distribution. Mann–Whitney *U* and chi-square were run to compare between metformin groups (No vs. Yes). Odds ratios (ORs) and 95% confidence intervals (CIs) were calculated by multivariate binomial logistic regression models with frailty status (nonfrail and frail) as the dependent variable, and metformin exposure (No vs. Yes) as the independent variable. Age, race, ethnicity, BMI, median household income, CCI, diabetes complications, duration of diabetes, use of insulin or sulfonylureas, level of glycemia control, and hospitalizations in the previous year were used as covariates in the multivariate analysis. These covariates were selected as they have been previously associated with frailty in multiple studies (Espinoza & Hazuda, 2008; Feng et al., 2017; Vetrano et al., 2019). We also analyzed nonfrail and frail subgroups by changing the cutoff for the FI status from ≥.21 to ≥.30. For the purposes of the analysis, frailty was dichotomized into nonfrail (including robust and prefrail) and frail. All analyses were performed using the SPSS, version 24.0 for Macintosh (SPSS, Inc., Chicago, Illinois) and SAS for Windows, version 3.71 (SAS Institute Inc., Cary, North Carolina). All statistical tests were two-tailed, and statistical significance was assumed for a *p* value < 0.05.

## Results

*Patient characteristics* (see Table 1): 763 participants had at least 30 variables or more needed for calculation of the FI: 56.7% White, 77.0% non-Hispanic, and the mean age was 72.9 (*SD* = 6.8) years. The proportion of robust, prefrail and frail patients was 2.9% (*n* = 22), 46.7% (*n* = 356) and 50.5% (*n* = 385), respectively. As seen in Table 1, 50.6% (*n* = 386) and 49.4% (*n* = 377) of the patients took or did not take metformin, respectively. Participants with frailty were more likely to have type 2 diabetes with complications, longer duration of type 2 diabetes, higher use of medications, higher use of insulin or sulfonylureas, and more comorbidities. Individuals with frailty were less likely to be married and to use metformin. We have also included in the Supplemental Materials a table of participant characteristics stratified according to metformin exposure.

**Table 1.** Participant Characteristics Stratified by Frailty Status.

Variable	Non-frail (*n* = 378, 49.5%)	Frail (*n* = 385, 50.5%)	Total (*n* = 763, 100%)	*p* value
Age, mean (*SD*)	72.40 (6.2)	73.33 (7.3)	72.87 (6.8)	.190
Male, *n* (%)	372 (98.4)	378 (98.2)	750 (98.3)	.805
Married, *n* (%)	210 (55.5)	158 (41.0)	368 (48.2)	**<.0001**
Caucasian *n* (%)	219 (57.9)	214 (55.6)	433 (56.7)	.512
Non-Hispanic, *n* (%)	284 (75.1)	304 (79.0)	588 (77.1)	.208
Median household income, $ (*SD*)	54,004 (25,068)	50,877 (22,883)	52,426 (24,026)	.100
BMI, mean (*SD*)	29.82 (5.2)	30.07 (6.0)	29.95 (5.6)	.542
Diabetes with end organ damage, *n* (%)^a^	107 (28.3)	140 (36.4)	247 (32.4)	**.017**
Duration of diabetes, y (*SD*)	8.46 (5.4)	9.48 (5.2)	8.97 (5.6)	**.009**
More than five medications, *n* (%)	344 (91.0)	380 (98.7)	724 (94.9)	**<.0001**
Metformin, *n* (%)	225 (59.5)	161 (41.8)	386 (50.6)	**<.0001**
Insulin or sulfonylurea, *n* (%)	189 (50.0)	221 (57.4)	410 (53.7)	**.040**
Glycemic control				
Tight (HbA1c ≤ 7), *n* (%)	219 (57.9)	202 (52.5)	421 (55.2)	.146
Intermediate (HbA1c > 7, <9), *n* (%)	128 (33.9)	137 (35.6)	265 (34.7)	
Poor (HbA1c ≥ 9), *n* (%)	31 (8.2)	46 (11.9)	77 (10.1)	
Frailty Index, mean (*SD*)	16 (.03)	.28 (.06)	0.22 (0.07)	**<.0001**
Charlson CI, mean (*SD*)	5.75 (1.65)	6.91 (2.00)	6.33 (1.93)	**<.0001**

*Note. n* = number of participants; BMI= body mass index; CI = confidence interval.

a Diabetes with end organ damage: Patients diagnosed with one or more of the following diagnosis: retinopathy, neuropathy, and nephropathy. Mann–Whitney *U* test (for non-normally distributed variables) and chi-square for continuous variables and categorical variables, respectively. Significant differences between metformin groups are in bold (*p* < .05).

### Metformin Exposure

There were significant differences in metformin exposure scores between the groups (Table 1). In binomial logistic regression, metformin exposure was negatively associated with frailty in unadjusted (OR = .49, 95% CI = .37–.65, *p* ≤ .001) and adjusted (OR = .55, 95% CI = .39–.77, *p* ≤ .001) models (Table 2). The analysis was repeated after excluding individuals with creatinine clearance <30 mL/min (*n* = 48). Metformin exposure was associated with frailty in unadjusted (OR = .57, 95% CI = .42–.77, *p* < .001) and adjusted (OR = .65, 95% CI = .46–.90, *p* = .011) analysis in the remaining 715 patients with a creatinine clearance ≥30 mL/min. When patients with heart failure (*n* = 60) were excluded from the analysis, metformin exposure was still associated with frailty in unadjusted (OR = .53, 95% CI = .39–.72, *p* < .001) and adjusted (OR = .58, 95% CI = .42–.82, *p* = .002) analysis in the remaining 703 patients without heart failure. When the analysis looked at the nonfrail and frail subgroups resulting from raising the FI cutoff from ≥.20 to ≥.25, metformin exposure was associated with frailty in unadjusted (OR = .51, 95% CI = .37–.71, *p* < .001) and fully adjusted models (OR = .59, 95% CI = .42–.85, *p* = .004).

**Table 2.** Effects of Metformin Exposure on Frailty in Older Veterans With Diabetes.

Metformin exposure	Unadjusted Odds Ratios (95% CI)	*p* value	Adjusted Odds Ratios (95% CI)	*p* value
No metformin		1 (reference)		
Metformin	.49 (.37–.65)	**<.001**	.55 (.39–.77)	**.001**

*Note.* Model was adjusted for age, race, ethnicity, and median household income, diabetes with end organ damage, duration of diabetes, use of insulin or sulfonylureas, level of glycemia control, Charlson Comorbidity Index, and for hospitalizations in the previous year. Significant associations are in bold (*p* < .05). CI = confidence interval.

## Discussion

Metformin has pleiotropic effects that include anti-inflammatory, antioxidant mechanisms and actions on insulin resistance, phenomena that are part of frailty and diabetes. As proposed in our hypothesis, metformin exposure was independently associated with a lower risk for frailty in a sample of older adults with type 2 diabetes. Metformin’s association with frailty still remained after excluding patients with either renal impairment or heart failure, absolute, and relative contraindications to the use of metformin or when the FI was raised.

The association between metformin exposure and lower risk for frailty may be explained by its pleiotropic actions on multiple pathways including insulin resistance, chronic low-grade inflammation, oxidative stress, stem cell dysfunction and mitochondrial dysfunction (Abdelhafiz et al., 2016; Garcia-Esquinas et al., 2015; Morley et al., 2014). In this study, a strong association between metformin exposure and frailty was identified, even after adjusting for covariates. Few observational studies have investigated the links of metformin exposure with frailty. A study in older veterans with type 2 diabetes examined whether metformin effects on survival were attenuated in those individuals with frailty. Compared with patients taking sulfonylureas, patients exposed to metformin had a lower mortality risk, but the effect was reduced among those identified as frail—suggesting that metformin may be more effective at preventing rather than treating frailty (Wang et al., 2014). In another study, investigators identified a cohort of older male veterans with diabetes who at baseline were found to be free from several medical and mental conditions commonly associated with frailty. Metformin exposure was associated with a decrease in the incidence of chronic conditions commonly associated with frailty, which the investigator inferred may lead to a lower incidence of frailty (Wang et al., 2017). Our main analysis differed from these two previous studies evaluating metformin’s effect on frailty. Unlike our study, neither study specifically evaluated the association of metformin exposure with frailty status. Another important difference is how frailty status was defined. Where we used an accepted conceptual framework for the evaluation of frailty status, both these studies relied primarily on the number of medical and psychological conditions in their attempt to define frailty. Metformin pleiotropic effects involving actions at many different levels may explain the associations we found in this cohort of older veterans with diabetes.

The retrospective design of our study may result in some bias. It is conceivable that individuals who were prescribed metformin or who took metformin are inherently different from those who did not take the drug because they were taking this oral agent for specific indications or contraindications. We tried to overcome these biases by using multivariate adjustments including several known covariates and analyzing subgroups according to the presence of chronic renal impairment, heart failure, or a higher frailty severity as the outcome. Only randomized controlled trials would allow us to make valid inferences of cause and effect regarding the efficacy of metformin at reducing frailty. However, these studies may not be feasible or even ethical in this population as metformin is now considered a first-line agent for the treatment of prediabetes and type 2 diabetes. Future prospective studies may also include older patients with type 2 diabetes who were newly prescribed metformin and matched controls who were prescribed other antidiabetic medications using propensity matching techniques. The possibility of implementing future controlled trials using metformin in patients without prediabetes or type 2 diabetes also deserves consideration as the benefits of this medication go beyond glucose control.

Strengths of this study include the large number of participants with complete data as well as the use of a validated process to calculate the FI incorporating comprehensive electronic health record data. There are a few limitations. We used a convenience instead of a randomly selected sample. The study was also limited to veterans at one medical center, and ethnic, racial, educational, and socioeconomic composition may be different from other facilities in the United States. Our study is cross-sectional, limiting our conclusions about the causal effect of metformin exposure on frailty. Nevertheless, our results and conclusions can have important clinical implications and encourage future studies.

Metformin has become the mainstay for the treatment of diabetes. The clinical implications of these findings are that metformin may be considered as therapy for older patients with diabetes, not only for diabetes control but possibly to improve the prognosis of frailty. As metformin is safe, well-tolerated, and inexpensive, this agent may also be effective for the prevention of frailty in older adults (Espinoza et al., 2020). Future research may examine the association of metformin exposure with frailty in older individuals as part of longitudinal studies.

## Conclusion

The study reveals that metformin exposure was associated with a lower risk for frailty after adjustment for covariates. Future research may examine differences between older individuals from more diverse samples and as part of longitudinal studies.

## Supplemental Material

Supplemental_material_1 – Supplemental material for Is There an Association Between Metformin Exposure and Frailty? Click here for additional data file. Supplemental material, Supplemental_material_1 for Is There an Association Between Metformin Exposure and Frailty? by Dhanya Baskaran, Raquel Aparicio-Ugarriza, Juliana Ferri-Guerra, Raneem Milyani, Hermes Florez and Jorge G. Ruiz in Gerontology and Geriatric Medicine

SUPPLEMENTARY_MATERIALS_2 – Supplemental material for Is There an Association Between Metformin Exposure and Frailty? Click here for additional data file. Supplemental material, SUPPLEMENTARY_MATERIALS_2 for Is There an Association Between Metformin Exposure and Frailty? by Dhanya Baskaran, Raquel Aparicio-Ugarriza, Juliana Ferri-Guerra, Raneem Milyani, Hermes Florez and Jorge G. Ruiz in Gerontology and Geriatric Medicine

## References

[bibr1-2333721420924956] Abdelhafiz A. H. Koay L. Sinclair A. J. (2016). The effect of frailty should be considered in the management plan of older people with type 2 diabetes. Future Science OA, 2(1). 10.4155/fsoa-2015-0016 PMC5137864 28031949

[bibr2-2333721420924956] Abdelhafiz A. H. Sinclair A. J. (2019). Cognitive frailty in older people with type 2 diabetes mellitus: The central role of hypoglycaemia and the need for prevention. Current Diabetes Reports, 19(4), Article 15. 10.1007/s11892-019-1135-4 30806826

[bibr3-2333721420924956] Barzilai N. Crandall J. P. Kritchevsky S. B. Espeland M. A. (2016). Metformin as a tool to target aging. Cell Metabolism, 23(6), 1060–1065. 10.1016/j.cmet.2016.05.011 27304507 PMC5943638

[bibr4-2333721420924956] Benjamin S. M. Wang J. Geiss L. S. Thompson T. J. Gregg E. W. (2015). The Impact of Repeat Hospitalizations on Hospitalization Rates for Selected Conditions Among Adults With and Without Diabetes, 12 US States, 2011. Preventing chronic disease, 12, E200–E200. 26583572 10.5888/pcd12.150274 PMC4655479

[bibr5-2333721420924956] Chang S. F. Lin H. C. Cheng C. L. (2018). The Relationship of Frailty and Hospitalization Among Older People: Evidence From a Meta-Analysis. Journal of Nursing Scholarship, 50(4), 383–391. doi:10.1111/jnu.12397 29874399

[bibr6-2333721420924956] Espinoza S. E. Hazuda H. P. (2008). Frailty in older Mexican-American and European-American adults: Is there an ethnic disparity? Journal of the American Geriatrics Society, 56(9), 1744–1749. 18662198 10.1111/j.1532-5415.2008.01845.x

[bibr7-2333721420924956] Espinoza S. E. Musi N. Wang C. P. Michalek J. Orsak B. Romo T. Powers B. Conde A. Moris M. Bair-Kelps D. Li Y. Ganapathy V. Jergensen E. J. Kelly L. C. Jiwani R. (2020). Rationale and study design of a randomized clinical trial of metformin to prevent frailty in older adults with pre-diabetes. The Journals of Gerontology, Series A: Biological Sciences & Medical Sciences, 75(1), 102–109. doi:10.1093/gerona/glz078 10.1093/gerona/glz078 PMC7175970 30888034

[bibr8-2333721420924956] Feng Z. Lugtenberg M. Franse C. Fang X. Hu S. Jin C. Raat H. (2017). Risk factors and protective factors associated with incident or increase of frailty among community-dwelling older adults: A systematic review of longitudinal studies. PLoS ONE, 12(6), e0178383. 28617837 10.1371/journal.pone.0178383 PMC5472269

[bibr9-2333721420924956] Ferri-GuerraJ.Aparicio-UgarrizaR.SalgueroD.BaskaranD.MohammedY. N.FlorezH.RuizJ. G. (2020). The association of frailty with hospitalizations and mortality among community dwelling older adults with diabetes. The Journal of Frailty & Aging, 9, 94–100.3225918310.14283/jfa.2019.31

[bibr10-2333721420924956] Fried L. P. Tangen C. M. Walston J. Newman A. B. Hirsch C. Gottdiener J. Seeman T. Tracy R. Kop W. J. Burke G. McBurnie M. A., & Cardiovascular Health Study Collaborative Research Group. (2001). Frailty in older adults: Evidence for a phenotype. The Journals of Gerontology, Series A: Biological Sciences & Medical Sciences, 56(3), M146–M156. 10.1093/gerona/56.3.m146 11253156

[bibr11-2333721420924956] Garcia-Esquinas E. Graciani A. Guallar-Castillon P. Lopez Garcia E. Rodriguez-Manas L. Rodriguez-Artalejo F. (2015). Diabetes and risk of frailty and its potential mechanisms: A prospective cohort study of older adults. Journal of the American Medical Directors Association, 16(9), 748–754. 10.1016/j.jamda.2015.04.008 25986874

[bibr12-2333721420924956] Liu Y. Sayam S. Shao X. Wang K. Zheng S. Li Y. Wang L. (2017). Prevalence of and Trends in Diabetes Among Veterans, United States, 2005-2014. Preventing chronic disease, 14, E135. 10.5888/pcd14.170230 PMC5737977 29240552

[bibr13-2333721420924956] Morley J. E. Malmstrom T. K. Rodriguez-Manas L. Sinclair A. J. (2014). Frailty, sarcopenia and diabetes. Journal of the American Medical Directors Association, 15(12), 853–859. 10.1016/j.jamda.2014.10.001 25455530

[bibr14-2333721420924956] Orkaby A.R. Nussbaum L. Ho Y.L. Gagnon D. Quach L. Ward R., et al The Burden of Frailty Among U.S. Veterans and Its Association With Mortality, 2002-2012. The Journals of Gerontology, Series A: Biological Sciences & Medicine Sciences. 2019;74(8):1257-64. 10.1093/gerona/gly232 PMC6625596 30307533

[bibr15-2333721420924956] Piskovatska V. Stefanyshyn N. Storey K. B. Vaiserman A. M. Lushchak O. (2019). Metformin as a geroprotector: Experimental and clinical evidence. Biogerontology, 20(1), 33–48. 10.1007/s10522-018-9773-5 30255224

[bibr16-2333721420924956] Rena G. Hardie D. G. Pearson E. R. (2017). The mechanisms of action of metformin. Diabetologia, 60(9), 1577–1585. 28776086 10.1007/s00125-017-4342-z PMC5552828

[bibr17-2333721420924956] Rockwood K. Mitnitski A. (2007). Frailty in relation to the accumulation of deficits. The Journals of Gerontology, Series A: Biological Sciences & Medical Sciences, 62(7), 722–727. 10.1093/gerona/62.7.722 17634318

[bibr18-2333721420924956] Searle S. D. Mitnitski A. Gahbauer E. A. Gill T. M. Rockwood K. (2008). A standard procedure for creating a frailty index. BMC Geriatrics, 8, Article 24. 10.1186/1471-2318-8-24 PMC2573877 18826625

[bibr19-2333721420924956] Sinclair A. J. Abdelhafiz A. Dunning T. Izquierdo M. Rodriguez Manas L. Bourdel-Marchasson I. Morley J. E. Munshi M. Woo J. Vellas B. (2018). An international position statement on the management of frailty in diabetes mellitus: Summary of recommendations 2017. Journal of Frailty & Aging, 7(1), 10–20. 10.14283/jfa.2017.39 29412437

[bibr20-2333721420924956] Sinclair A. J. Rodriguez-Manas L. (2016). Diabetes and frailty: Two converging conditions? Canadian Journal of Diabetes, 40(1), 77–83. 10.1016/j.jcjd.2015.09.004 26683240

[bibr21-2333721420924956] Vetrano D. L. Palmer K. Marengoni A. Marzetti E. Lattanzio F. Roller-Wirnsberger R. Samaniego L. L. Rodríguez-Mañas L. Bernabei R. Onder G., & Joint Action ADVANTAGE WP4 Group. (2019). Frailty and multimorbidity: A systematic review and meta-analysis. Journal of Gerontology, Series A: Biological Sciences & Medical Sciences, 74, 659–666. 10.1093/gerona/gly110 29726918

[bibr22-2333721420924956] Wang C. P. Lorenzo C. Espinoza S. E. (2014). Frailty attenuates the impact of metformin on reducing mortality in older adults with type 2 diabetes. Journal of Endocrinology, Diabetes & Obesity, 2(2), 1031. PMC4264048 25506599

[bibr23-2333721420924956] Wang C. P. Lorenzo C. Habib S. L. Jo B. Espinoza S. E. (2017). Differential effects of metformin on age related comorbidities in older men with type 2 diabetes. Journal of Diabetes and Its Complications, 31(4), 679–686. 10.1016/j.jdiacomp.2017.01.013 28190681 PMC5654524

