# Supplementary material for: Is There an Association Between Metformin Exposure and Frailty?
Source: Gerontol Geriatr Med. 2020 Jun 15;6:2333721420924956. doi: 10.1177/2333721420924956 (PMC7297486; doi:10.1177/2333721420924956)
Supplement: Supplemental_material_1 – Supplemental material for Is There an Association Between Metformin Exposure and Frailty? [file Supplemental_material_1.pdf]

SUPPLEMENTARY MATERIALS 1

Frailty Index (44-item)

**Demographics-Social**
1. Marital Status
2. VA Service Period
3. VA Service Connection
4. Religion
**Medical Conditions**
5. Congestive heart failure
6. Peripheral vascular disease
7. Coronary disease
8. COPD
9. Stroke
10. Hypertension
11. Atrial Fibrillation
12. Arthritis
13. Osteoporosis
14. Cancer
15. OSA

**N of Medications**
16. > 5

**Psychological**

17. Dementia
18. Depression
19. Psychosis
20. Anxiety
21. PTSD
22. Alcohol abuse
23. Smoking
24. Substance Abuse

**Laboratories**
25. White blood cells
26. Hemoglobin
27. Platelets
28. Sodium
29. Potassium
30. Chloride
31. BUN
32. GFR
33. Glucose
34. CO_2

**Vitals and Measures**
35. BMI
36. SBP

37. DBP
**Functional**
38. Bathing
39. Toileting
40. Feeding
41. Dressing
42. Transferring
43. Incontinence

**Sensory problems**
44. Hearing loss
