# Supplementary material for: Is There an Association Between Metformin Exposure and Frailty?
Source: Gerontol Geriatr Med. 2020 Jun 15;6:2333721420924956. doi: 10.1177/2333721420924956 (PMC7297486; doi:10.1177/2333721420924956)
Supplement: SUPPLEMENTARY_MATERIALS_2 – Supplemental material for Is There an Association Between Metformin Exposure and Frailty? [file SUPPLEMENTARY_MATERIALS_2.pdf]

SUPPLEMENTARY MATERIALS 2

Participant Characteristics Stratified by Metformin exposure.

**Variable**	**No Metformin (n= 377, 49.4%)**	**Metformin (n= 386, 50.6%)**	**Total (n=763, 100%)**	**p-value**
Age (Mean, SD)	74.4 (7.6)	71.3 (5.5)	72.9 (6.8)	0.154
Male	373 (49.7)	377 (50.3)	750 (98.3)	0.175
Married	187 (50.8)	181 (49.2)	368 (48.2)	0.206
White	215 (49.7)	218 (50.3)	433 (56.7)	0.88
Non-Hispanic	277 (52.9)	311 (37.1)	588 (77%)	**0.02**
Median Household Income (Mean, SD)	50503 (23715)	54304 (24208)	52426 (24026)	0.113
BMI (Mean, SD)	29.4 (5.6)	30.5 (5.5)	30.0 (5.6)	**0.009**
DM With Organ Damage*	128 (51.8)	119 (48.2)	247 (32.4)	0.357
Duration of Diabetes	9.3 (5.4)	8.6 (5.2)	9.0 (5.3)	0.093
More than 5 Meds	358 (49.4)	366 (50.6)	724 (94.9)	0.929
Insulin or Sulfonylurea	205 (50)	205 (50)	410 (53.7)	0.725
Glycemic Control				
Tight (HbA1C<=7)	219 (52)	202 (48.0)	421 (55.2)	0.229
Adequate (HbA1C>7)	120 (45.3)	145 (54.7)	265 (34.7)	
Poor (HbA1C>=9)	38 (49.4)	39 (50.7)	77 (10.1)	
CCI_Age (Mean, SD)	6.69 (2.01)	5.99 (1.78)	6.34 (1.93)	**<0.001**

n = number of participants; SD = standard deviation; BMI= body mass index. *Diabetes with End organ damage: patients diagnosed with one or more of the following diagnosis: retinopathy, neuropathy and nephropathy.

Mann-Whitney U (for non-normally distributed variables) and Chi-Square for continuous variables and categorical variables, respectively. Significant differences between metformin groups are in bold ($p < .05$).
